# Study on the relationship between homocysteine and general metabolic indexes in healthy population in Hebei Province, China

**DOI:** 10.3389/fendo.2025.1523157

**Published:** 2025-08-13

**Authors:** Hongru Feng, Xiaoliang Wang, Lili Yu, Qianqian Zheng, Zhaoqi Wang

**Affiliations:** ^1^ Department of Physical Examination Center, The Second Hospital of Hebei Medical University, Shijiazhuang, China; ^2^ Department of Neurosurgery, The Second Hospital of Hebei Medical University, Shijiazhuang, China; ^3^ Department of Neurology, The Second Hospital of Hebei Medical University, Shijiazhuang, China; ^4^ Department of Metabolic Regulation, Shinshu University School of Medicine, Matsumoto, Japan

**Keywords:** red blood cells, hyperhomocysteinemia, high-density lipoprotein, healthy population, risk factor

## Abstract

**Background:**

The aging problem is a significant issue and challenge currently faced by the whole world. Hyperhomocysteinemia (HHcy) is a common phenomenon among the older adult. Increasing evidence suggests a link between HHcy and multiple systemic issues in the older adult-related diseases. Therefore, the identification of high-risk factors for HHcy in a healthy screening population can effectively regulate the occurrence, progression of HHcy, thereby reducing the incidence of older adult-related diseases.

**Methods:**

A total of 10,511 individuals who underwent a comprehensive health examination at the Second Hospital of Hebei Medical University, China from 2021 to 2022 were included. Data on gender, age, carotid ultrasound(CCA), blood pressure(BP), body mass index(BMI), serum levels of homocysteine(Hcy), total cholesterol(TC), triglycerides(TG), low-density lipoprotein(LDL-C), high-density lipoprotein(HDL-C), alanine aminotransferase(ALT), aspartate aminotransferase(AST), red blood cells(RBC), creatinine(Cr), urea, uric acid(UA), fasting blood-glucose(FBG), and glycated hemoglobin(GHb) concentration were collected.

**Results:**

Hyperhomocysteinemia was associated with gender, age, BP, CCA, BMI, elevated levels of TC, TG, LDL-C, Cr, urea, UA, as well as decreased levels of HDL-C and RBC. Among these factors male, above 65 years old age, hypertension, carotid artery abnormalities, UA, and Cr were identified as independent risk factors, while HDL-C and RBC were identified as protective factors.

**Conclusion:**

The prevalence of HHcy was very high during routine physical examination especially the senior citizens in Hebei Province, China. Therefore, high-risk populations should be the focus of public health policies, and strengthening early intervention can reduce the occurrence of HHcy, thereby delaying the onset and progression of older adult-related diseases.

## 1 Introduction

Homocysteine (Hcy), an endogenous amino acid, which can be metabolised enzymatically to either be converted into cysteine or to be remethylated back into methionine (1). Since McCully’s (2) groundbreaking discovery in 1969 highlighted the role of Hcy in altering cases of atherosclerosis, Hcy has emerged as a recognized biomarker and an independent associated with various diseases. Its association with conditions such as coronary artery disease, cardiovascular disease, cerebrovascula disease (3–7), neurocognitive impairment (8, 9), depression, and osteoporotic fractures in the older adult (10, 11) has attracted significant attention from researchers and clinicians. However, to date, the majority of Hcy researches have focused on the correlation between Hcy concentration and diseases, with limited emphasis on epidemiological investigations. Therefore, the prevalence of HHcy in routine health examinations conducted in Hebei, were assessed, with an aim to elucidate the correlation between HHcy and diseases. The associations of Hcy with gender, age, carotid ultrasound, BP, BMI, serum levels of TC, TG, LDL-C, HDL-C, ALT, AST, RBC, Cr, urea, UA, FBG, and GHb concentration were investigated. These findings provide valuable evidence for the prevention and management of HHcy and its associated diseases.

## 2 Participants

Screen out 10,511 individuals who underwent health check-ups at the Second Hospital of Hebei Medical University from 2021 to 2022 were included in this study. The data collected for these individuals included gender, age,carotid ultrasound, BP,BMI,serum levels of Hcy, TC, TG, LDL-C, HDL-C, ALT, AST, RBC, Cr, urea, UA, FBG, and GHb concentration. Exclusion criteria for individuals affecting Hcy results included recent use of lipid-lowering drugs, folic acid, B-vitamins, and other relevant factors. The study population excluded individuals with severe cardiovascular and cerebrovascular diseases, kidney diseases, thyroid diseases, malignant tumors, as well as those with incomplete information or test results. This study was approved by the Ethics Committee of the Second Hospital of Hebei Medical University. The informed consent to participate was waived by the Ethics Committee of the Second Hospital of Hebei Medical University. All methods were carried out according to relevant guidelines and regulations.

## 3 Method

### 3.1 Detection method

Carotid ultrasound results were completed by attending physicians and above, and gender, age, body mass index and blood pressure results were completed by nurses with nursing qualifications. Blood samples were collected in the morning after fasting and were subsequently analyzed for serum red blood cell parameters using a DxH 800 Sysmex blood analyzer. Glycosylated hemoglobin, homocysteine, total cholesterol, triglycerides, low-density lipoprotein, high-density lipoprotein, alanine aminotransferase, aspartate aminotransferase, creatinine, fasting blood glucose, urea, and uric acid were measured using a Cobas 8000 automatic analyzer for biochemical analysis.

### 3.2 Diagnostic criteria

The following definitions are used in this study. HHcy: Serum Hcy concentration ≥15 μmol/L according to the “Guidelines” China cerebrovascular prevention and treatment. red blood cell concentration: male <3.80 10^12^/L, female <3.80 10^12^/L; Blood uric acid concentration: male >428 μmol/L, female >357 μmol/L; Creatinine concentration: Male >97 μmol/L, female >73 μmol/L; Urea concentration: Male >8.0 mmol/L, female >7.5 mmol/L; Dyslipidemia: triglyceride concentration ≥1.70 mmol/L, serum TC concentration ≥6.10 mmol/L, serum LDL-C concentration ≥3.37 mmol/L, serum HDL-C male <1.04 mmol/L; High GLU: fasting GLU concentration >6.0 mmol/L; Hemoglobin A1c >6.0%; Alanine transaminase: male >50U/L, female >40U/L; Aspartate transaminase: male >40U/L, female >35U/L. Carotid artery abnormalities: carotid internal membrane thickening and plaque formation.

### 3.3 Data analysis

“All statistical analyses were performed using SPSS 26.0 software. Continuous variables were expressed as mean and standard deviation, while categorical variables were presented as frequency and percentage. The independent samples t-test was employed to compare the gender, age,CCA, BP,BMI,serum levels of TC,TG, LDL-C, HDL-C, ALT, AST, RBC, Cr, urea, UA, FBG, and GHb concentration between HHcy group and non-HHcy group. The differences in carotid artery status and blood pressure between the two groups were compared using the chi-square test. The indicators with statistical significance for comparison between groups were selected for stepwise logistic regression analysis to screen for risk factors. Finally, the indicators with statistical significance were the risk factors. A P-value <.05 was considered statistically significant.

#### 3.3.1 Comparison of serum Hcy concentration and prevalence of HHcy in different subjects

**Figure 1:**
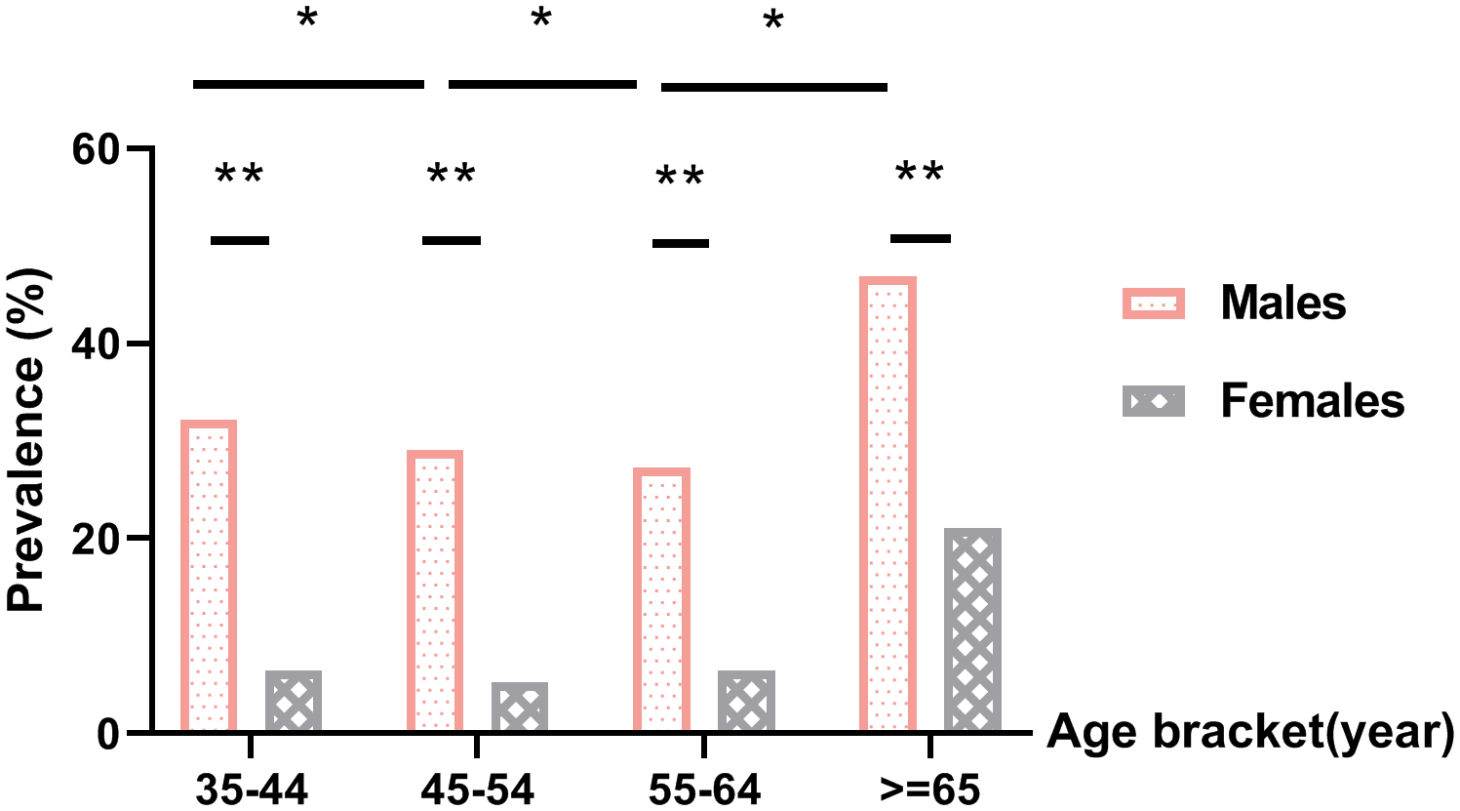
Prevalence of hyperhomocysteinemia. *P<.05 for comparison of HHcy prevalence between different age groups within the same gender, **P<.05 for comparison of HHcy prevalence between males and females in the same age bracket.

The overall prevalence of HHcy among participants was 22% (18.1% in males and 3.9% in females). The detection rate of HHcy in females was lower than that in males with a statistically significant difference (χ2 = 963.174, P <.05). In each age group (35–44 years old, 45–54 years old, 55–64 years old, 65 years old and older), the detection rate of HHcy in males was higher than that in females (P <.05), as shown in **Table 1**. The plasma Hcy levels all showed a trend of decreasing first and then increasing, Among them, the HHcy detection rate was the highest in the group aged 65 years and above (P <.05), as shown in **Figure 1**.

**Table 1:** Prevalence of hyperhomocysteinemia.

Age bracket	Total			Males			Females		
	n	Number of HHcy subjects	Prevalence (%)	n	Number of HHcy subjects	Prevalence (%)	n	Number of HHcy subjects	Prevalence (%)
35-44	2590	537	17.2	888	420	32.1	1702	117	6.4
45-54	2487	542	17.9	1145	467	29.0	1342	75	5.3
55-64	1751	417	19.2	975	364	27.2	776	53	6.4
≥65	1370	817	37.4	732	647	46.9	638	170	21.0
Total	8198	2313	22.0	3740	1898	33.7	4458	415	8.5

HHcy, hyperhomocysteinemi.

#### 3.3.2 Comparison of index mean between HHcy group and non-HHcy group

The CCA, BP, BMI and serum concentrations of UA, urea, Cr, TC, TG, and LDL-C were significantly higher in the HHcy group compared to the non-HHcy group. Conversely, HDL-C and RBC concentrations were significantly lower in the HHcy group than in the non-HHcy group (P<.05). Serum concentrations of FBG, GHb, ALT and AST in HHcy group were not significant compared with those in non-HHcy group, as shown in **Table 2**.

**Table 2:** Comparisons of CCA, BP, BMI, and serum concentrations of LDL-C, TG, TC, HDL-C, ALT, ASL, RBC, Cr, KFPTT, Ur, UA and GHb between HHcy group with non-HHcy group.

Influential factors	Non-HHcy	HHcy	t/*χ* ^2^	P
CCA [n (%)]			*χ* ^2^ = 227.366	<0.001
1	4503 (84.0)	860 (16.0)		
2	3695 (71.8)	1453 (28.2)		
BP (mmHg) [n (%)]			*χ* ^2^ = 208.398	<0.001
1	5808 (82.1)	1270 (17.9)		
2	2390 (69.6)	1043 (30.4)		
BMI	24.86 ± 3.4	25.97 ± 3.48	*t*=-13.762	<0.001
TC (mmol/L)	5.01 ± 1.00	4.91 ± 1.00	*t*=4.040	<0.001
TG (mmol/L)	1.54 ± 1.17	1.69 ± 1.26	*t*=-5.580	<0.001
HDL-C (mmol/L)	1.37 ± 0.31	1.26 ± 0.28	*t*=15.625	<0.001
LDL-C (mmol/L)	2.92 ± 0.85	2.87 ± 0.85	*t*=2.871	0.004
ALT (mmol/L)	23.35 ± 27.49	24.07 ± 18.87	*t*=-1.178	0.239
AST (mmol/L)	23.99 ± 18.16	24.68 ± 13.18	*t*=-1.720	0.086
RBC (10^12^/L)	4.64 ± 0.43	4.77 ± 0.47	*t*=-12.708	<0.001
Cr (μmol/L)	70.50 ± 13.22	83.57 ± 25.74	*t*=-33.071	<0.001
FBG (mmol/L)	5.58 ± 1.47	5.62 ± 1.35	*t*=-1.206	0.228
Urea (mmol/L)	4.63 ± 1.12	5.12 ± 1.62	*t*=-16.764	<0.001
UA (μmol/L)	324.2 ± 84.14	368.95 ± 91.02	*t*=-22.175	<0.001
GHb (%)	5.90 ± 0.82	5.92 ± 0.76	-1.053	0.292

Non-HHcy, normal homocysteine; HHcy, hyperhomocysteinemia; CCA, carotid ultrasound (normal =1, carotid internal membrane thickening and plaque formation =2); BP, blood pressure(normal=1, hypertension =2); BMI, body mass index; TC, total cholesterol; TG, triglycerides; HDL-C, high-density lipoproteincholesterol; LDL-C, low-density lipoprotein cholesterol; ALT, alanine aminotransferase; AST, aspartate aminotransferase; RBC, red blood cell; Cr, Creatinine; KBG, fasting blood-glucose; Urea, urea; UA, uric acid; GHb, glycated hemoglobin.

#### 3.3.3 Binary logistic regression analysis of HHcy-related risk factors

The results indicated that male, carotid internal membrane thickening and plaque formation,hypertension and of 65 years old and older age along with elevated UA, Cr levels are risk factors for HHcy; whereas elevated HDL-C levels and RBC count serve as protective factors against HHcy, as shown in **Table 3**.

**Table 3:** Hyperhomocysteinemia related risk factors analyzed by bivariate nonconditional logistic regression model.

Related risk factors	SE	Warld	P	OR (95%CI)
Gender				
mal (control)	–	–	–	–
femal	0.083	195.287	<0.001	0.313 (0.266-0.368)
Age (y)				
35-44	–	115.151	<0.001	–
45-54	0.073	5.365	0.021	0.844 (0.731-0.974)
55-64	0.08	8.126	0.004	0.795 (0.680-0.931)
≥65	0.079	41.74	<0.001	1.669 (1.429-1.950)
CCA				
1 (normal)	–	–	–	–
2	0.053	138.936	<0.001	1.871 (1.686-2.076)
HDL-C (mmol/L)	0.097	13.922	<0.001	0.697 (0.576-0.842)
RBC (10^12^/L)	0.071	7.732	0.005	0.820 (0.713-0.943)
Cr (mmol/L)	0.002	191.194	<0.001	1.033 (1.028-1.038)
UA (mmol/L)	0.000	4.601	0.032	1.001 (1.000-1.001)
BP (mmol/L)				
1 (normal)	–	–	–	–
2	0.057	8.581	0.003	1.181 (1.057-1.321)

SE, standard error; OR, odds rato; CCA, carotid ultrasound; HDL-C, high-density lipoproteincholesterol; RBC, red blood cell; Cr, Creatinine; UA, uric acid; BP, blood pressure.

## 4 Discussion

Studies have demonstrated a significant association between Hcy levels and the occurrence of cardiovascular and cerebrovascular diseases, as well as the severity of cognitive impairment. In the Framingham study, there was a positive correlation observed between Hcy levels and both overall mortality and cardiovascular mortality among older adult individuals. Furthermore, a dose-dependent relationship exists between Hcy levels and the development of CSVD, with elevated homocysteine concentrations being linked with cognitive dysfunction (12, 13). That is, for every 3umol/L increase in plasma Hcy, the incidence of ischemic heart disease increases by 11% (14), and for every 2.5umol/L increase in plasma Hcy concentration, the risk of stroke increases by approximately 20% (15). For every 5umol/L increase in Hcy, a patient’s risk of AD increases by 40%, and individuals with Hcy levels higher than 14umol/L are twice as likely to develop AD 8 years later (16). With the aging problem in China, the older adult are facing great threat of aging-related chronic diseases. Therefore, the investigation of Hcy metabolism in Hebei is bound to play a very important role in the prevention and treatment of cardiovascular and cerebrovascular diseases in middle-aged and older adult people.

In this study, a total of 10,511 individuals who participated in the health examination in the Hebei area were included. The overall prevalence of HHcy in the healthy population was found to be 22.0% (18.1% among men and 3.9% among women). Previous reports have indicated that HHcy incidence in China ranges from 10% to 68% (17, 18,) with higher rates observed among older adult individuals and males, which is consistent with our findings. Gender differences may be attributed to variations in muscle mass, estrogen status, lifestyle factors, and vitamin levels (19). Furthermore, it has been established that homocysteine production is associated with creatinine-creatinine synthesis (20). Males generally exhibit a higher muscle mass, leading to an increasing demand for creatine biosynthesis and subsequently resulting in elevated Hcy production (21). Hormonal disparities between genders may also contribute to sex-related differences. Furthermore, compared to females, Chinese men have a higher prevalence of alcohol consumption and smoking habits, both of which are positively correlated with Hcy concentration (22). Alternatively, discrepancies in folate, vitamin B12, and vitamin B6 status between the sexes could partially account for the observed gender difference (19).

Age is a associated with HHcy; Xu (23) et al.’s study confirmed its effect on the prevalence of HHcy while our study demonstrated that men had significantly higher rates of HHcy than women across different age groups.The plasma levels of Hcy exhibited a pattern of initial decline followed by an increase in both males and females. Bivariate unconditional logistic regression analysis revealed that age was a associated with HHcy. This could potentially be attributed to altered renal function, decreased vitamin levels, and impaired renal metabolism of Hcy (24–27).

Furthermore, Hcy has been found to interfere with hepatic lipid metabolism leading to disorders in lipid metabolism. Liao (28) et al.’s study demonstrated that CBS-/-/apoE-/- mice with hyperhomocysteinemia exhibited an increased incidence of atherosclerosis along with reduced HDL levels. In this study, the analysis of the biochemical results revealed that the HHcy group exhibited lower levels of HDL-C compared to the normal group, and a negative correlation was observed between HHcy and HDL-C. Consistent with these findings, each unit increase in HDL-C was associated with a 0.697 times decrease in the risk of HHcy, indicating that HDL-C serves as “good cholesterol” in blood lipids and its reduction is negatively correlated with the risk of atherosclerosis. UA and Cr are crucial indicators for assessing renal function. This study identified UA and Cr as risk factors for HHcy. Malinow et al. (29) proposed that the metabolic product of Hcy was the precursor of UA synthesis. It may be related to the effect of Hcy on renal function and its metabolic pathway and oxidative stress function (30). Xu Shan (31) et al. found that there was a certain correlation between the production and metabolism of plasma Hcy and blood uric acid in the study of the prevalence of hyperuricemia in patients with H-type hypertension. At the same time, because the kidney is also involved in the filtration and metabolism of Hcy, the damage of kidney function will also lead to the increase of Hcy, and there is an interaction between kidney function and Hcy level, which is consistent with the results of this study. Li et al. (32) reported that a high serum UA concentration can acgtivate proinflammatory factors,damage vascular endothelial function,increase oxidative stress,and lead to renal inadequacy.They also reported that Hcy accumulation in the plasma strengthens lipid peroxidation,which further influences kidney function,and Hcy and UA show synergistic effects.

Erythrocytes are the site of homocysteine production. This study found that for each unit increase in red blood cells, the risk of HHcy decreased by 0.820 times, and the mechanism is not clear. Previous research has confirmed a positive correlation between CIMT and plasma Hcy levels (33, 34), with the mechanism involving endothelial injury, oxidative stress, and alterations in lipid metabolism (35). The results of this study are consistent with those findings. Clinical studies have found that the number of individuals with elevated plasma Hcy levels is 2.8 times higher in hypertensive patients compared to the normal population, and high Hcy hyperhomocysteinemia is a associated with hypertension (36). Wilcken’s (37) research also suggests a link between hypertension and gene mutations in MTHFR. In this experiment, a close relationship was found between a history of hypertension and Hcy levels, with both hypertensive cerebrovascular disease patients and the control group exhibiting higher plasma Hcy levels than those without a history of hypertension. Multiple linear regression analysis revealed that hypertension is a associated with HHcy. Simultaneously, a plethora of evidence indicates that hyperhomocysteinemia and hypertension are both crucial and modifiable risk factors for cardiovascular and cerebrovascular events, particularly stroke. The coexistence of these two conditions exponentially increases the risk of cardiovascular and cerebrovascular events. Effectively controlling plasma homocysteine levels in hypertensive individuals may be a pivotal strategy to address the high incidence of stroke.

Li Y, et al.’s study found a close association between HHcy and blood glucose levels as well as transaminase levels (38–41). However, significant differences in blood glucose, glycated hemoglobin, and transaminase between the two population groups have not been detected in this study. This may be due to the uneven distribution in the stratified sampling of subjects or the insignificant impact of blood glucose, glycated hemoglobin, and transaminase on Hcy levels.

## 5 Conclusion

In summary, the prevalence of HHcy was high in Hebei province, China. Male, above 65 years old age, hypertension, carotid artery abnormalities, elevated Cr, UA, decreased HDL-C and RBC constitute high-risk factors for HHcy. Adopting a rational lifestyle, timely blood pressure reduction and weight management, and implementing effective intervention measures can contribute to lowering HCY levels and thereby reducing the incidence of older adult-related diseases.

## Data Availability

The original contributions presented in the study are included in the article/supplementary material. Further inquiries can be directed to the corresponding author.
